# Multicenter evaluation of a lateral-flow device test for diagnosing invasive pulmonary aspergillosis in ICU patients

**DOI:** 10.1186/s13054-015-0905-x

**Published:** 2015-04-17

**Authors:** Susanne Eigl, Juergen Prattes, Michaela Lackner, Birgit Willinger, Birgit Spiess, Mark Reinwald, Brigitte Selitsch, Michael Meilinger, Peter Neumeister, Frederike Reischies, Albert Wölfler, Reinhard B Raggam, Holger Flick, Stephan Eschertzhuber, Robert Krause, Dieter Buchheidt, Christopher R Thornton, Cornelia Lass-Flörl, Martin Hoenigl

**Affiliations:** Section of Infectious Diseases and Tropical Medicine, Department of Internal Medicine, Medical University of Graz, Auenbruggerplatz 15, A- 8036 Graz, Austria; Division of Pulmonology, Department of Internal Medicine, Medical University of Graz, Auenbruggerplatz 15, 8036 Graz, Austria; Division of Hygiene and Medical Microbiology, Medical University of Innsbruck, Schoepfstraße 41/III, 6020 Innsbruck, Austria; Division of Clinical Microbiology, Department of Laboratory Medicine, Medical University of Vienna, Währinger Gürtel 18-20, 1090 Vienna, Austria; Department of Hematology and Oncology, Mannheim University Hospital, University of Heidelberg, Theodor-Kutzer-Ufer 1 – 3, 68167 Mannheim, Germany; Division of Hematology, Department of Internal Medicine, Medical University of Graz, Auenbruggerplatz 38D, 8036 Graz, Austria; Clinical Institute of Medical and Chemical Laboratory Diagnostics, Medical University of Graz, Auenbruggerplatz 15, 8036 Graz, Austria; Department of Anesthesia and Intensive Care Medicine, Innsbruck Medical University, Anichstraße 35, 6020 Innsbruck, Austria; Biosciences, College of Life and Environmental Sciences, University of Exeter, Stocker Road, EX4 4QD Exeter, UK; Division of Infectious Diseases, Department of Medicine, University of California, 200 West Arbor Drive #8208, San Diego, CA 92103 USA

## Abstract

**Introduction:**

The incidence of invasive pulmonary aspergillosis (IPA) in intensive care unit (ICU) patients is increasing, and early diagnosis of the disease and treatment with antifungal drugs is critical for patient survival. Serum biomarker tests for IPA typically give false-negative results in non-neutropenic patients, and galactomannan (GM) detection, the preferred diagnostic test for IPA using bronchoalveolar lavage (BAL), is often not readily available. Novel approaches to IPA detection in ICU patients are needed. In this multicenter study, we evaluated the performance of an *Aspergillus* lateral-flow device (LFD) test for BAL IPA detection in critically ill patients.

**Methods:**

A total of 149 BAL samples from 133 ICU patients were included in this semiprospective study. Participating centers were the medical university hospitals of Graz, Vienna and Innsbruck in Austria and the University Hospital of Mannheim, Germany. Fungal infections were classified according to modified European Organization for Research and Treatment of Cancer/Mycoses Study Group criteria.

**Results:**

Two patients (four BALs) had proven IPA, fourteen patients (sixteen BALs) had probable IPA, twenty patients (twenty-one BALs) had possible IPA and ninety-seven patients (one hundred eight BALs) did not fulfill IPA criteria. Sensitivity, specificity, negative predictive value, positive predictive value and diagnostic odds ratios for diagnosing proven and probable IPA using LFD tests of BAL were 80%, 81%, 96%, 44% and 17.6, respectively. Fungal BAL culture exhibited a sensitivity of 50% and a specificity of 85%.

**Conclusion:**

LFD tests of BAL showed promising results for IPA diagnosis in ICU patients. Furthermore, the LFD test can be performed easily and provides rapid results. Therefore, it may be a reliable alternative for IPA diagnosis in ICU patients if GM results are not rapidly available.

**Trial registration:**

ClinicalTrials.gov NCT02058316. Registered 20 January 2014.

## Introduction

Invasive pulmonary aspergillosis (IPA) caused by the ubiquitous environmental mold *Aspergillus* is a leading cause of morbidity and mortality in immunocompromised patients, including those in intensive care units (ICUs) [1]. Well-known risk factors for IPA (for example, prolonged neutropenia, stem cell or solid organ transplant (SOT)) are present in a significant number of patients who develop IPA in the ICU [2]. However, IPA has also been reported in ICU patients lacking classical risk factors [3-5], including those with chronic obstructive pulmonary disease (COPD), systemic use of corticosteroids, advanced liver disease, severe sepsis, septic shock and acute renal failure [5-8]. Although researchers in a previous study reported an incidence of about 5.8% [9], the true incidence of IPA in the ICU remains unknown.

Timely use of antifungal drugs significantly decreases IPA-related mortality in high-risk patients [10]; thus, early diagnosis is a crucial cornerstone of successful IPA management. Early diagnosis remains a challenge, however, as clinical signs and symptoms and diagnostic imaging are often non-specific. Also, conventional culture methods lack sensitivity, and tissue biopsy is invasive and rarely possible in critically ill patients. IPA diagnosis is particularly challenging in ICU patients without underlying hematologic malignancies. First, chest X-rays and computed tomographic images are often difficult to interpret, as many of them show pulmonary infiltrates, atelectasis or signs of preexisting lung tissue damage [11]. Second, diagnostic *Aspergillus* galactomannan (GM) determination in serum seems less reliable in non-neutropenic patients than in patients with neutropenia, as the angioinvasive growth of *Aspergillus* in non-neutropenic patients is typically confined by the local immune response in lung tissues. Therefore, the level of circulating GM in the bloodstream may be too low to be detected [12,13].

Detection of GM in bronchoalveolar lavage (BAL) fluid and conventional microbiological culture of BAL fluid samples are the current preferred tests for IPA diagnosis in ICU patients [3,14,15]. GM is a polysaccharide cell wall component of *Aspergillus* species that is released during active growth. Using a commercially available enzyme-linked immunosorbent assay, GM can be detected in BAL fluid, blood, urine and cerebrospinal fluid [16-18]. However, GM detection has some limitations. Factors such as comedications (for example, β-lactam antibiotics), underlying diseases, host factors (for example, renal failure and/or renal replacement therapy), radiologic findings and clinical signs have to be considered for the correct interpretation of GM levels [19-22]. One of the crucial limitations of the GM test is the need for appropriately equipped laboratories where the test can be performed by trained staff, leading to variable turnaround times, which can be up to several days [14].

Many of these limitations are overcome by the *Aspergillus*-specific lateral-flow device (LFD) test, a novel and rapid single-sample test for IPA diagnosis. Because of its simplicity, the LFD test can be performed in rudimentary facilities with minimal staff training. Patient BAL fluid samples do not need pretreatment, which means that test results can be available within 10 to 15 minutes of sample receipt. This point-of-care test uses a monoclonal antibody (mAb JF5) to detect an extracellular mannoprotein antigen that is secreted exclusively during active growth of *Aspergillus* species [23,24]. LFD test results are read by the naked eye, and they have previously been shown to be reproducible between different studies and different laboratories [25]. Previous studies have shown the remarkable diagnostic potential of the LFD test in diagnosing IPA using BAL fluid samples [26-30], but data on the performance of the LFD test in ICU patients are limited, with only two previous single-center studies of subsets of ICU patients [29,30]. In this multicenter study, we evaluated the LFD test using BAL fluid specimens for early diagnosis of IPA in ICU patients.

## Materials and methods

This multicenter cohort study was performed at the three Austrian medical university hospitals (in Graz, Innsbruck and Vienna) and the University Hospital of Mannheim, Germany. The study comprised 149 previously unpublished BAL fluid samples obtained from 133 ICU patients with clinical suspicion of IPA who were tested routinely for the presence of *Aspergillus* species between January 2010 and June 2014. The decision whether to obtain BAL samples and send them for mycological workup was completely up to the treating physician’s discretion. Patients with SOT were excluded from the analysis. Samples at the Medical University Hospital of Graz, Austria (n = 70), the Medical University Hospital of Vienna, Austria (n = 18) and the University Hospital of Mannheim, Germany (n = 10), were included prospectively between February 2012 and June 2014. In Vienna, samples were included only if they had a positive *Aspergillus* culture result. Patients at the Medical University Hospital of Innsbruck were included, in part, prospectively (n = 31; January 2013 to June 2013). In Innsbruck, another 20 samples were tested retrospectively for patients who had been included in the Innsbruck fungal infection biobank sample collection between 2010 and 2012 (samples frozen at −70°C). Data of this manuscript were presented previously in poster presentations at three conferences: the Sixth Advances Against Aspergillosis Meeting 2014 [31], the 2014 MYK meeting [32], and IDWeek 2014 [33]. Other than that no data of patients included in this study have been published previously.

Patients were classified as having proven, probable, possible or no IPA in accordance with slightly revised criteria of the European Organization for Research and Treatment of Cancer/Mycoses Study Group (EORTC/MSG) [34], with the inclusion of ICU stay above 4 days as a host factor [15,16]. Modifications were necessary because host factors in these guidelines were originally defined for patients with hematologic malignancies, and a significant proportion of ICU patients with IPA do not fulfill these host factors [35]. This was shown by Blot and colleagues, with EORTC/MSG host factors being absent in approximately 30% of proven IPA cases [35]. In previous studies, the median (interquartile range (IQR)) time from ICU admission to development of IPA was between 2 days (IQR, 1 to 9) and 4 days (IQR, 1 to 8) [35,36]. Therefore, we decided to introduce ICU stay above 4 days as an additional host factor in our modified EORTC/MSG criteria. In addition, we evaluated test performance when classifying IPA according to the predefined EORTC/MSG criteria (without ICU stay as a host factor) [34] and a previously published clinical algorithm [35], instead of our modified EORTC/MSG criteria.

Depending on where the patient was included in the study, LFD testing was performed at the Microbiology Laboratory, Department of Internal Medicine, Medical University of Graz; the Institute of Hygiene and Microbiology, Innsbruck Medical University; the Division of Clinical Microbiology, Medical University of Vienna, Austria; or the scientific laboratory, Department of Hematology and Oncology, Mannheim University Hospital, Germany. For LFD testing, 100 μl of untreated BAL fluid samples were applied to the LFD. The results were read by the naked eye and interpreted after a 15-minute incubation at room temperature, as recommended previously [23]. Bound antigen–antibody–gold complexes were recorded as a red line with intensity proportional to the antigen concentration. Test line intensity ranged from strong positive (+++) to weak positive (+) or negative (−) [25] and depended on the antigen contents of BAL fluid samples. Regardless of the intensity of the test lines, all positive results indicated germination of spores and existence of hyphae in the lungs and were therefore interpreted as positive. Test results were compared with routinely performed BAL GM tests, direct microscopic and culture results.

Statistical analysis was performed using SPSS version 22 software (IBM, Armonk, NY, USA). The diagnostic performance of the LFD test for probable or proven IPA versus no IPA (putative or proven IPA when using the alternative clinical algorithm) were evaluated, and negative predictive value (NPV), positive predictive value (PPV), sensitivity and specificity were calculated. Additionally, diagnostic odds ratios (DORs) with 95% confidence intervals (95% CIs) were determined.

This study was conducted in accordance with the Declaration of Helsinki (1996), Good Clinical Practice and applicable local regulatory requirements and law. The study protocol was approved by the local ethics committee at the Medical University Graz, Austria (EC number 25-221 ex 12/13), as well as by the ethics committees of the Medical University of Vienna (EC number 1656/2013) and the Medical University of Innsbruck (EC number UN 4926), and the trial is registered at ClinicalTrials.gov (identifier NCT02058316). As clinical data of patients treated at the Mannheim University Hospital were analyzed retrospectively with a scientific intent and data were concurrently obtained pseudonymized, approval by the local ethics committee (Faculty of Medicine in Mannheim Ethics Committee) was not required according to the German ethics committee regulations. All data presented have been deidentified and are therefore not attributable to individual patients. The ethics committees waived written informed consent of participating patients. The performance evaluation of a medical product was also reported to the Austrian Agency for Health and Food Safety (protocol number INS-621000-0478).

## Results

A total of 149 BAL fluid samples from 133 patients were included in the final analysis. All patients were treated in an ICU setting at the time bronchoscopy was performed. The median age of the study population was 60 years (range, 19 to 85), and 65.4% (n = 87) were men and 34.6% (n = 46) were women. The demographic characteristics and underlying diseases of the study population are shown in Table 1. Known risk factors for IPA were present in the majority of the study population, and these included COPD (32 (21.5%) of 149;), acute leukemia (18 (12%) of 149), neutropenia (18 (12%) of 149), chronic systemic corticosteroid use (11 (7%) of 149), bone marrow transplantation (9 (6%) of 149), bronchial carcinoma (8 (5%) of 149), influenza A viral pneumonia (7 (5%) of 149) and liver cirrhosis and/or alcoholic hepatitis (6 (4%) of 149).

**Table 1:** **Demographic data and underlying diseases of intensive care unit patients with bronchoalveolar lavage lateral-flow-device test results** ^**a**^

	**Present study population**	**Graz**	**Innsbruck**	**Vienna**	**Mannheim**
Number of BALs/patients	149/133	70/61	51/49	18/13	10/10
Sex					
Male	87 (65.4%)	45 (51.7%)	28 (32.2%)	6 (6.9%)	8 (9.2%)
Female	46 (34.6%)	16 (34.8%)	21 (45.7%)	7 (15.2%)	2 (4.3%)
Median age (range)	60 (19 to 85)	66 (19 to 83)	58 (26 to 85)	59.5 (41 to 74)	55.5 (40 to 75)
Type of ICU admission (BALs)					
Medical	121/149 (81.2%)	60/70 (85.7%)	35/51 (68.6%)	16/18 (88.9%)	10/10 (100%)
Neurologic	6/149 (4%)	5/70 (2.9%)	1/51 (2%)	–	–
Elective surgery	2/149 (1.3%)	–	1/51 (2%)	1/18 (5.6%)	–
Emergency surgery	8/149 (5.4%)	4/70 (5.7%)	4/51 (7.8%)	–	–
Trauma	12/149 (8.1%)	1/70 (1.4%)	10/51 (19.6%)	1/18 (5.6%)	–
Primary underlying disease (BALs)					
Pulmonary disease	44/149 (29.5%)	21/70 (30%)	14/51 (27.5%)	6/18 (33.3%)	3/10 (30%)
Heart disease	25/149 (16.8%)	17/70 (24.3%)	4/51 (7.8%)	4/18 (22.2%)	–
Hematologic malignancies	19/149 (12.8%)	8/70 (11.4%)	3/51 (5.9%)	2/18 (11.1%)	6/10 (60%)
Trauma	12/149 (8.1)	1/70 (1.4%)	10/51 (19.6%)	1/18 (5.6%)	–
Neurologic disease	14/149 (9.4%)	11/70 (15.7%)	3/51 (5.9%)	–	–
Gastrointestinal disease	13/149 (8.7%)	3/70 (4.3%)	7/51 (13.7%)	3/18 (16.7%)	–
Metabolic disease	4/149 (2.7%)	1/70 (1.4%)	3/51 (5.9%)	–	–
Other malignancies	5/149 (3.4%)	3/70 (4.3%)	2/51 (3.9%)	–	–
Rheumatologic disease	4/149 (2.7%)	3/70 (4.3%)	–	–	1/10 (10%)
Others	9/149 (6%)	2/70 (2.9%)	5/51 (9.8%)	2/18 (11.1%)	–
IPA classification					
Proven IPA (BALs/patients)	4/2	–	–	3/1	1/1
Probable IPA (BALs/patients)	16/14	6/6	8/7	2/1	–
Possible IPA (BALs/patients)	21/20	6/5	6/6	–	9/9
No IPA (BALs/patients)	108/97	58/50	37/36	13/11	–

^a^BAL, Bronchoalveolar lavage; COPD, Chronic obstructive pulmonary disease; IPA, Invasive pulmonary aspergillosis.

Our classification of IPA in accordance with the modified EORTC/MSG diagnostic criteria is depicted in Table 1. Overall, 12% (16 of 133) of patients (20 (13.4%) of 149 BALs) had probable or proven IPA. Both patients with proven IPA died, one of whom had acute myeloid leukemia and had undergone recent allogeneic stem cell transplant and the other of whom had acute-on-chronic liver failure and a prolonged ICU stay. As the primary underlying disease among the patients with probable IPA, four had underlying COPD (one with inherited immunodeficiency and three with systemic corticosteroid treatment), four septic pneumonia (including two with influenza A viral pneumonia), three had underlying hematologic malignancies, two had liver cirrhosis and/or alcoholic hepatitis and one had trauma. The majority of patients with probable IPA (9 of 14 patients, 11 of 16 BALs) did fulfill EORTC/MSG host factors, whereas 5 of 14 patients (5 of 16 BALs) with probable IPA fulfilled only the newly introduced host factor of ICU stay above 4 days (all of them had ICU stays of 10 days or longer). GM testing was performed in ten BAL fluid samples of patients with probable IPA, and the results were positive in nine of those (two of those BALs also yielded a positive culture for *Aspergillus* spp.). Cultures for *Aspergillus* were also positive in the remaining 7 of the 16 BAL fluid samples of patients with probable IPA; GM evaluation was not performed in 6 of these 7 samples.

The sensitivity and specificity of the BAL LFD test for diagnosing probable or proven IPA (versus no IPA) in our cohort were 80% and 81%, respectively, when we used the modified EORTC/MSG classification (and 87% and 81%, respectively, when we used predefined EORTC/MSG criteria). The LFD test performance for probable or proven IPA versus no IPA (and putative or proven IPA versus no IPA) is depicted in Table 2. The sensitivity of the BAL LFD test in samples with a positive GM test was 89% (eight of nine samples had positive LFD results). In samples of probable cases (according to modified EORTC/MSG criteria) with a positive culture but without GM evaluation, the sensitivity was 75% (six of eight samples had a positive LFD result).

**Table 2:** ***Aspergillus*** **-specific lateral-flow device test performance for diagnosing probable or proven versus no invasive pulmonary aspergillosis** ^**a**^

	**Sensitivity**	**Specificity**	**PPV**	**NPV**	**DOR (95% CI)**
Modified EORTC/MSG criteria [34]^b^					
Overall study population	80% (16/20)	81% (88/108)	44% (16/36)	96% (88/92)	17.6 (5.3 to 58.3)
Graz	83% (5/6)	79% (46/58)	29% (5/17)	98% (46/47)	19.2 (2.0 to 179.9)
Innsbruck	75% (6/8)	95% (35/37)	75% (6/8)	95% (35/37)	52.5 (6.2 to 447.6)
Vienna	80% (4/5)	54% (7/13)	40% (4/10)	88% (7/8)	4.7 (0.4 to 54)
Mannheim^c^	100% (1/1)	78% (7/9)	33% (1/3)	100% (7/7)	9 (0.3 to 200)
Original revised EORTC/MSG criteria [34]					
Overall study population	87% (13/15)	81% (101/124)	36% (13/36)	98% (101/103)	28.5 (6 to 135)
Clinical algorithm according to Blot *et al*. [35]					
Overall study population	83% (10/12)	79% (108/137)	26% (10/39)	98% (108/110)	18.6 (3.9 to 89.7)

^a^CI, Confidence interval; DOR, Diagnostic odds ratio; EORTC/MSG, European Organization for Research and Treatment of Cancer/Mycoses Study Group; NPV, Negative predictive value; PPV, Positive predictive value. ^b^Including intensive care unit stay longer than 4 days as a newly introduced host factor and bronchoalveolar lavage fluid galactomannan >0.5. ^c^Results from Mannheim represent *Aspergillus*-specific lateral-flow device test performance for probable or proven versus possible or no invasive pulmonary aspergillosis (IPA), owing to the fact that only proven and possible cases were reported by this center. Classification was carried out according to EORTC/MSG criteria with and without modifications (possible IPA cases were excluded), and proven or putative IPA versus no IPA was classified according to the clinical algorithm published by Blot and colleagues [35].

Positive LFD test results were found in 20 (19%) of 108 BAL fluid samples from 18 patients who did not fulfill modified EORTC/MSG IPA criteria. In 6 (30%) of these 20 BAL fluid samples, the patients (n = 6) had mycological evidence of mold infection. Conventional BAL cultures grew *Aspergillus* species in all six cases, and one patient had a positive BAL culture, and a highly positive BAL GM level (optical density index (ODI) = 5.77) and a positive serum GM level; however, this patient did not fulfill radiological and clinical criteria for IPA. Most false-positive LFD tests (n = 14) revealed only a weak positive result (+). The LFD test results were negative in another six BAL fluid samples from four patients who did not fulfill IPA criteria, but these samples showed growth of various *Aspergillus* species in culture. In addition, the LFD test results were positive in 3 (14%) of 21 BAL fluid samples (from 3 patients) with possible IPA. False-negative LFD results were observed in 4 (20%) of 20 BALs (from 4 patients) with probable or proven IPA (3 of 16 BALs from patients with probable IPA and 1 of 4 with proven IPA). In the patient with proven IPA, three consecutive BAL samples were obtained within 1 month. Whereas the LFD test results were positive in the first two BAL samples, the result was negative in the third (under systemic antimold treatment) while the culture remained positive. In one of the three patients with probable IPA and false-negative results, mycological evidence was provided by positive BAL GM (ODI = 0.72); in the two remaining cases, evidence was provided by positive BAL culture. (GM testing was not performed in these latter two cases).

A comparison of the performance of the LFD test with conventional culture (which was performed in all cases) for probable or proven IPA versus no IPA according to modified EORTC/MSG criteria is shown in Table 3. In addition, BAL GM testing was performed in 53 of 149 BALs, for which the results were positive (ODI > 0.5) in 12 (23%) of the samples. BAL GM results were positive in 9 of 10 patients with probable or proven IPA and in 3 (7%) of 43 samples from patients without IPA.

**Table 3:** **Comparison of** ***Aspergillus*** **-specific lateral-flow device test and conventional bronchoalveolar lavage culture performance for diagnosing probable or proven versus no invasive pulmonary aspergillosis** ^**a**^

**Probable or proven IPA versus no IPA**	**LFD**	**Conventional BAL culture**
Sensitivity	80% (16/20)	50% (10/20)
Specificity	81% (88/108)	85% (88/103)
PPV	44% (16/36)	40% (10/25)
NPV	96% (88/92)	90% (88/98)
DOR (95% CI)	17.6 (5.3 to 58.3)	5.9 (2.1 to 16.5)

^a^BAL, Bronchoalveolar lavage; CI, Confidence interval; DOR, Diagnostic odds ratio; IPA, Invasive pulmonary aspergillosis; LFD, *Aspergillus*-specific lateral-flow device test; NPV, Negative predictive value; PPV, Positive predictive value. According to European Organization for Research and Treatment of Cancer/Mycoses Study Group criteria [34], probable IPA cases require mycological evidence by positive *Aspergillus* spp BAL culture, cytology, microscopy or positive galactomannan tests, in addition to host factors and clinical criteria.

## Discussion

We performed a multicenter study to evaluate the *Aspergillus* LFD test for early IPA diagnosis using BAL samples in a mixed cohort of ICU patients. The results across the four participating hospitals show that the LFD test provides accurate and rapidly available results for disease detection, with sensitivities and specificities of about 80% that were consistent when we used three different classification criteria for IPA. The advantages of the LFD test, which include rapid turnaround time (results available within 15 minutes) without the need for specialized laboratory equipment and highly trained staff, make it a promising alternative to the GM enzyme immunoassay, which is often not readily available immediately and can therefore delay early initiation of antifungal therapy.

Invasive fungal infections are an increasing threat in ICU patients. In Europe, reported incidence rates of IPA among ICU patients vary widely (0.2% to 6.9%) [9,11,37,38]. When analyzing only the proportion of patients with routine bronchoscopy and mycological workup that were included prospectively in our study cohort, the prevalence of probable or proven IPA was 11%. The higher rate may be explained by the fact that only high-risk patients (with routine mycological workup ordered by the treating physicians) were included in this study [39].

Previously published studies have shown the high diagnostic potential of the LFD test for IPA using human BAL fluid and serum samples in non-ICU patients [23,24,26,27,30,40]. In patients with SOT, LFD tests of BAL specimens showed promising diagnostic performance, with a sensitivity of 91% and a specificity of 83% [28]. Thus, the performance of the LFD in patients with SOT was comparable to the current gold standard of IPA diagnosis (GM testing), which has a sensitivity of 93% and a specificity of 89% in patients with SOT [41]. Similar diagnostic accuracy of the LFD was reported in a mixed population of immunocompromised patients, where sensitivity reached 80% and specificity was 95% [29]. In particular, the combination of a positive LFD and/or GM test for IPA diagnosis was very promising (sensitivity 90%, specificity 93%) [29]. Evaluation of the LFD test in patients with underlying pulmonary diseases yielded a sensitivity of 77% and specificity of 90% [30].

Two of the previous studies on the LFD test included ICU patients [29,30]. One study included 104 ICU patients with underlying pulmonary diseases [30], and the other include 9 ICU patients, 5 with underlying hematologic malignancies and 4 of whom had sepsis. Summarizing the results in the ICU patients of those two studies (13 patients with probable or proven IPA), the LFD test had a sensitivity of 77%, a specificity of 90% and a DOR of 28.5 (95% CI, 6.6 to 123.2). The overall performance of the LFD in ICU patients throughout different studies highlights the great potential of this point-of-care test (sensitivity of 79%, specificity of 85% and NPV of 96%). In particular, the remarkably high 96% or greater NPV of the LFD test in ICU patients may make it a helpful tool to prevent overtreatment, which has become frequent in the ICU setting, as reported by Azoulay and colleagues [42]. Similarly high NPVs have also been reported in other studies of the LFD test in other patient groups [27-29].

Sensitivities (75% to 100%) and specificities (78% to 95%) were found to be relatively consistent over all four participating hospitals, with the exception of Vienna, where specificity was markedly lower (54%). The fact that the test’s performance was less convincing in Vienna than in the other centers might be explained by the differing inclusion criteria: In Vienna, only samples that grew molds were included, and most of those originated from patients without evidence of IPA. In addition, a significant proportion of samples included in Innsbruck were biobank samples from patients with mostly probable IPA. Different inclusion criteria, together with small sample sizes (in particular of probable or proven IPA cases) may explain differing PPVs, NPVs and DORs across the four study sites. Not surprisingly, PPVs were lower and NPVs higher in those settings (Graz and Mannheim) where ICU patients undergoing bronchoscopy with mycological workup were included prospectively than in Vienna and Innsbruck, where additional biobank samples and/or preselected samples with mold growth were included. PPVs and NPVs calculated for Graz and Mannheim may therefore be more applicable to a real-life medical ICU settings than those in Innsbruck and Vienna may. Nevertheless, we want to emphasize the value of adding Innsbruck biobank samples and mold culture–positive samples from Vienna for validation of sensitivity of this new test for this rare disease.

False-positive results occurred in 20 patients without IPA according to slightly modified EORTC/MSG criteria. As the EORTC/MSG criteria are focused on patients with hematologic malignancies and are not entirely appropriate for ICU patients without hematologic diseases, they may lead to an underestimation of IPA occurrence in these patients. Therefore, it is conceivable that a proportion of these false-positive cases might in fact have resulted from early stages of IPA at the times the bronchoscopies were performed.

Bergeron and colleagues previously reported that, in patients with hematologic malignancies, a mycological diagnostic strategy should be based on underlying disease and the leukocyte count [13]. Whereas patients with neutropenia usually develop angioinvasive forms of IPA, non-neutropenic patients tend to develop airway-invasive forms. As a consequence, serum biomarkers tend to give a false-negative result in critically ill non-neutropenic patients, and diagnostic BAL should be performed in suspected cases of IPA. This is in accordance with data previously published by Meersseman and colleagues, who reported a sensitivity of 88% for positive BAL culture for GM in ICU patients, but a sensitivity of 42% for serum GM in the same patient cohort [3]. Thus, bronchoscopy and BAL in non-neutropenic patients with suspected IPA seems to be crucial, and, in settings where GM results are not rapidly available, the LFD tests on BAL specimens might be a very promising alternative because in eight of nine of our cases with true-positive BAL GM results, the LFD test also yielded a positive result.

Consistent with our previously published data, we show in the present study that the performance of culture is limited by low sensitivity [10]. In the present study, the sensitivity of mycological culture reached 50%. However, in view of the increasing resistance of *Aspergillus* strains to antifungal drugs [43], mycological culture is essential for susceptibility testing and may allow detection of other molds (for example, the Mucorales) that are not detectable by GM or LFD.

Our study has several limitations, including the need for modifications of the EORTC/MSG revised definitions of 2008 with the inclusion of ICU stay above 4 days as a host factor. Modifications were necessary because host factors in these definitions were originally developed for severely immunocompromised patients, but they have not been evaluated in other non-neutropenic patients at risk for IPA, as described in the Methods section. Although we assume that our modified criteria might be more appropriate for the ICU population, and even though all patients who met definitions of probable IPA had ICU stays of 10 days and longer, the introduction of “ICU stay above 4 days” as a host factor (although based on previously published data) might be considered arbitrary. Therefore, we also calculated LFD test performance with predefined EORTC/MSG criteria (without ICU stay as a host factor) [34] and a previously published clinical algorithm (which is mainly limited by the fact that it requires positive *Aspergillus* culture for putative IPA cases, which has a sensitivity below 50%) [35], and we found comparable test performance. Another limitation is that GM antigen detection in BAL fluid, which seems to be the most promising diagnostic tool for IPA diagnosis in ICU patients [3], was not performed in every case. The use of positive culture results alone as a mycological criterion may have led to misclassification of IPA in some cases, which may explain the slight variance of LFD sensitivities among the participating centers. (The sensitivity of LFD was higher in those with a positive GM than in those with positive culture but without GM evaluation.) Also, whereas LFD testing of 87% of included samples was performed prospectively, testing of biobank samples from Innsbruck was performed retrospectively. Previous studies have shown, however, that BAL LFD test results may be valid when tested with previously frozen samples [27-29,44]. Furthermore, demographic data such as underlying diseases, as well as the proportion of proven or probable IPA, varied among the participating centers, which may limit the assessment of the LFD test’s performance.

## Conclusions

The use of the LFD test in BAL fluid specimens represents a promising diagnostic approach for IPA detection in ICU patients, with sensitivity and specificity comparable to previously published data for ICU BAL GM testing. Thus, the LFD test may be viewed as a credible alternative diagnostic tool in critically ill patients at risk for IPA in settings where GM evaluations are not rapidly available. In such settings, the LFD test may facilitate initiation of early antifungal therapy in patients with IPA. Future studies should also be done to evaluate the performance of the LFD test in bronchial secretion and sputum samples, which have recently been reported to be equally useful for IPA diagnosis [45].

## Key message

- The LFD test performed in BAL specimens may be a promising alternative to the current gold standard test (GM antigen detection) in ICU patients.

## Abbreviations

BAL: Bronchoalveolar lavage
CI: Confidence interval
COPD: Chronic obstructive pulmonary disease
DOR: Diagnostic odds ratio
EORTC: European Organization for Research and Treatment of Cancer
GM: Galactomannan
ICU: Intensive care unit
IPA: Invasive pulmonary aspergillosis
IQR: Interquartile range
LFD: *Aspergillus*-specific lateral-flow device
MSG: Mycoses Study Group
NPV: Negative predictive value
ODI: Optical density index
PPV: Positive predictive value
SOT: Solid organ transplant
